# The prevalence and impact of lysogeny among oral isolates of *Enterococcus faecalis*

**DOI:** 10.1080/20002297.2019.1643207

**Published:** 2019-07-25

**Authors:** Roy H. Stevens, Hongming Zhang, Christine Sedgley, Adam Bergman, Anil Reddy Manda

**Affiliations:** a Kornberg School of Dentistry, Temple University, Philadelphia, PA, USA; b Department of Endodontology, Oregon Health and Science University, Portland, OR, USA

**Keywords:** *Enterococcus faecalis*, bacteriophage, prophage, lysogeny, phage ΦEf11

## Abstract

Bacterial phenotypic properties are frequently influenced by the uptake of extrachromosomal genetic elements, such as plasmids and bacteriophage genomes. Such modifications can result in enhanced pathogenicity due to toxin production, increased toxin release, altered antigenicity, and resistance to antibiotics. In the case of bacteriophages, the phage genome can stably integrate into the bacterial chromosome as a prophage, to produce a lysogenic cell. Oral enterococcal strains have been isolated from subgingival plaque and the root canals of endodontically-treated teeth that have failed to heal. Previously, we isolated a bacteriophage, phage ɸEf11, induced from a lysogenic *Enterococcus faecalis* strain recovered from the root canal of a failed endodontic case. PCR analysis using phage ɸEf11-specific oligonucleotide primers, disclosed that lysogens containing ɸEf11 prophages were commonly found among oral *E. faecalis* strains, being detected in 19 of 61 (31%) strains examined. Furthermore, in comparison to an isogenic cured strain, cultures of a lysogen harboring an ɸEf11 prophage exhibited altered phenotypic characteristics, such as increased persistence at high density, enhanced biofilm formation, and resistance to a bacteriophage lytic enzyme. From these results we conclude that lysogeny is common among oral *E. faecalis* strains, and that it alters properties of the lysogenic cell.

The uptake of mobile genetic elements, such as plasmids and bacteriophage (phage) genomes, can have a profound impact upon the host bacterial cell acquiring them. Acquired properties that increase population fitness, improve survival and confer virulence can all be the result of the activity of the products of genes located on these elements [1–3]. *Enterococcus faecalis* is an organism that is infrequently (prevalence ≈ 1%) found in the oral cavity of healthy individuals [4], but is transiently recovered following the consumption of certain foods, such as cheeses [5,6]. However, *E. faecalis* is found more frequently (≈ 5%) in the subgingival plaque of patients with adult periodontal disease [7], and is the most frequently recovered organism (prevalence ≈ 70–78%) from the filled root canals of endodontically-treated teeth that have failed to heal [8–10]. Previous reports have indicated that the incidence of plasmids among oral *E. faecalis* isolates may be as high as 81% [11]. However, there is little information available on the incidence of acquired phage genomes (in the form of lysogen prophages) among oral *E. faecalis* strains. In a previous article, we reported that phage could be induced from 4 out of 10 *E. faecalis* isolates obtained from the oral cavity/infected root canals [12]. However, due to the limited sample size of that investigation (n = 10), it is not possible to draw any meaningful conclusions regarding the prevalence of lysogeny among *E. faecalis* of the oral cavity. Furthermore, the methodology used in that work (phage induction and plaque assay) was intended for the isolation of bacteriophages from oral *E. faecalis* strains, not to screen for lysogeny among *E. faecalis* strains. Consequently, this would not have been the most sensitive or effective approach for detecting prophages in the *E. faecalis* strains. Now that sequence data are available for a temperate phage infecting an oral *E. faecalis* strain [13], it is possible to use PCR to screen a large panel of oral strains for the presence of the prophage. In the present investigation screening 61 oral *E. faecalis* strains, we present PCR evidence that lysogeny is indeed prevalent among oral *E. faecalis* strains, and that the presence of a prophage has altered the properties of the host cell.

## Methods and materials

### Microorganisms

The source and relevant characteristics of the 61 oral *E. faecalis* strains used in this study are listed in Table 1. All strains were grown in Brain Heart Infusion Broth^@^ at 37°C in stationary cultures. In addition, we included a panel of unrelated bacterial species to test the specificity of the primers that we used to detect the ɸEf11 sequence. These included: *Streptococcus mutans, S. sanguis, Staphylococcus aureus, Finegoldia (Peptostreptococcus) magna (magnus), Clostridium perfringens, Actinomyces israelii*, and *Eggerthella (Eubacterium) lenta (lentum)*.

**Table 1.** *E. faecalis* strains used and presence/absence of ɸEf11 ORF43.

Strain	Source	Antibiotic status	ORF 43 Present	Reference or Origin
*E. faecalis* TUSoD1	Root canal	Em^(r)^, TC^r^		a
*E. faecalis* TUSoD2	Root canal	Em^(r)^	**+**	a
*E. faecalis* TUSoD3	Root canal	Em^(r)^	**+**	a
*E. faecalis* TUSoD9	Root canal	Em^(r)^,TC^r^	**+**	a
*E. faecalis* TUSoD10	Root canal	Em^(r)^		a
*E. faecalis* TUSoD11	Root canal	Em^(r)^,TC^r^	**+**	a
*E. faecalis* TUSoD12	Root canal	TC^r^	**+**	a
*E. faecalis* TUSoD15	Root canal	TC^r^	**+**	a
*E. faecalis* TUSoD17	Root canal			a
*E. faecalis* TUSoD18	Root canal			a
*E. faecalis* GS1	Root canal			b
*E. faecalis* GS2	Root canal		**+**	b
*E. faecalis* GS3	Root canal			b
*E. faecalis* GS4	Root canal			b
*E. faecalis* GS6	Root canal			b
*E. faecalis* GS7	Root canal			b
*E. faecalis* GS8	Root canal	TC^r^	**+**	b
*E. faecalis* GS9	Root canal	TC^r^	**+**	b
*E. faecalis* GS10	Root canal			b
*E. faecalis* GS12	Root canal			b
*E. faecalis* GS13	Root canal			b
*E. faecalis* GS14	Root canal			b
*E. faecalis* GS15	Root canal			b
*E. faecalis* GS16	Root canal			b
*E. faecalis* GS17	Root canal			b
*E. faecalis* GS18	Root canal			b
*E. faecalis* GS19	Root canal			b
*E. faecalis* GS21	Root canal			b
*E. faecalis* GS22	Root canal		**+**	b
*E. faecalis* GS23	Root canal		**+**	b
*E. faecalis* GS24	Root canal	TC^r^		b
*E. faecalis* GS25	Root canal		**+**	b
*E. faecalis* GS26	Root canal			b
*E. faecalis* GS27	Root canal			b
*E. faecalis* GS28	Root canal			b
*E. faecalis* GS29	Root canal		**+**	b
*E. faecalis* GS30	Root canal	TC^r^		b
*E. faecalis* GS31	Root canal	TC^r^		b
*E. faecalis* GS32	Root canal			b
*E. faecalis* GS33	Root canal		**+**	b
*E. faecalis* GS34	Tongue			c
*E. faecalis* AA-OR3	Oral	Cl^(r)^, Cm^r^		d
*E. faecalis* AA-OR4	Oral	Cm^(r)^, TC^r^		d
*E. faecalis* AA-OR26	Oral	Cl^(r)^, Cm^r^, Em^r^, Gm^r^, TC^r^		d
*E. faecalis* AA-OR34	Oral	Cl^(r)^, Cm^(r)^	**+**	d
*E. faecalis* AA-T4	tongue	Cm^(r)^, TC^r^		d
*E. faecalis* AA-T26	Tongue	Cm^r^, Em^r^, Gm^r^, TC^r^		d
*E. faecalis* OS16	Oral			e
*E. faecalis* OS25	Tongue			e
*E. faecalis* E1	Oral isolate	Cl^r^,Cm^r^, Em^r^, TC^(r)^		f
*E. faecalis* E2	Oral isolate	Amp^(r)^, Cl^r^, Cm^r^, Em^(r),^TC^r^	**+**	f
*E. faecalis* E3	Oral isolate	Cl^r^, Em^(r)^	**+**	f
*E. faecalis* E4	Oral isolate	Cl^r^, Em^(r),^Tc^r^	**+**	f
*E. faecalis* E5	Oral isolate	Cl^r^	**+**	f
*E. faecalis* E6	Oral isolate	Cl^r^		f
*E. faecalis* E7	Oral isolate			f
*E. faecalis* E8	Oral isolate	Cl^r^, Tc^r^		f
*E. faecalis* E10	Oral isolate	Amp^(r)^, Cl^r^, Tc^r^		f
*E. faecalis* E11	Oral isolate	Cl^r^		f
*E. faecalis* ER3/25	Root canal			c
*E. faecalis* ER5/1	Root canal			c

r = resistant, (r) = intermediate resistance

Amp = ampicillin, Cm = chloramphenicol, Cl = clindamycin, Em = erythromycin, Gm = gentamicin, Tc = tetracycline

a-Stevens et al. 2009 [12], b-Sedgley et al. 2005a [11], c-Johnson et al. 2006 [79], d-Sedgley et al. 2006 [80], e-Sedgley et al. 2005b [81], f-Sedgley et al. 2004 [4].

### Primers and PCR conditions

**Figure 1.**
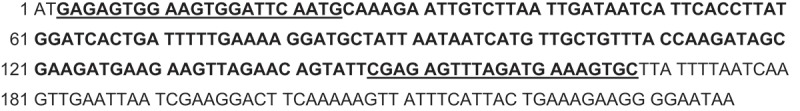
Phage ɸEf11 ORF43 sequence (237 bp). Primer binding sites are underlined. The 165 bp PCR amplicon product is shown in **bold** type.

Our previous sequencing of the genome of ɸEf11, a bacteriophage induced and isolated from an *E. faecalis* strain (TUSoD11) recovered from an infected root canal, demonstrated a genome of 42,822 bp distributed among 65 open reading frames (ORFs) [13]. Within that genome, ORF43 was designated as coding for a ‘hypothetical protein’. Our search of several databases failed to disclose any genes homologous to ɸEf11 ORF43. The sequence uniqueness of this target gene provided specificity of PCR amplicons produced by ORF43-specific primers, for the presence of the ɸEf11 DNA. ɸEf11 ORF43-specific oligonucleotide primers [forward (ɸEf11 F) and reverse (ɸEf11 R)] were designed which were predicted to produce a 165 bp amplicon in PCRs with phage ɸEf11 DNA templates (Table 2, Figure 1). Template DNA was prepared by suspending cells of each strain in lysis buffer [1% (v/v) Triton X-100, 20 mM Tris-HCl (pH 8.5), 2 mM EDTA], heating to 100°C for 10 min., and then recovering the released DNA in the supernatant following centrifugation. PCR mixtures contained: 5 μl (= 5 nmol) each of forward (ɸEf11 F) and reverse (ɸEf11 R) primer, 5 μl of DNA template solution (≈ 85ng DNA), 20 μl 2 x GoTaq PCR master mix (Promega), and 5 μl dH_2_O. In addition to the ORF43-specific forward and reverse oligonucleotide primers, an *E. faecalis* species-specific primer set (forward: 1F, reverse: 1R), and a universal primer set (forward: RRN4, reverse: RRN5) were used in control PCRs (Table 2). The *E. faecalis*-specific primers [14] were used as positive controls in PCRs for all the *E. feacalis* strains tested. Similarly, the universal primer set, which recognizes two highly conserved regions of eubacterial *16S rRNA* genes [15], was used as an internal positive control in PCRs involving DNA templates from non-enterococcal bacterial species. Additional (control) PCRs were prepared using *E. faecalis*-specific (1F and 1R) or universal primers (RRN4 and RRN5) instead of the phage ɸEf11 ORF43-specific primers. PCR conditions for reactions containing the ɸEf11 ORF43-specific primers (ɸEf11F and ɸEf11R) and *E. faecalis*-specific primers (1F and 1R) were: 97°C for 1min, followed by 26 cycles of (i) 94°C for 1 min, (ii) 50°C for 45 sec, and (iii) 72°C for 1 min. This was followed by an additional 4 min at 72°C. For PCRs utilizing the universal primers (RRN4 and RRN5), the reaction conditions were: 97°C for 1 min, followed by 25 cycles of (i) 95°C for 30 sec, (ii) 55°C for 30 sec and (iii) 72°C for 30 sec, followed by an additional 4 min at 72°C. Following PCR, amplification products were detected by agarose [2%(w/v)] gel electrophoresis and ethidium bromide staining.

**Table 2.** Primer sets used for PCR amplification.

Primer Set	Sequence	Purpose	Predicted Amplicon Size	Reference
ɸEf11 F ɸEf11 R	5ʹ-GAGAGTGGAAGTGGATTCAATG-3ʹ 5ʹ-GCACTTTCATCTAAACTCTCG-3’	Amplification of ɸEf11 ORF 43	165 bp	This study
1F 1R	5ʹ-GTTTATGCCGCATGGCATAAGAG-3ʹ 5ʹ-CCGTCAGGGGACGTTCAG-3’	*E. faecalis*-specific primers	310 bp	a
RRN4 RRN5	5ʹ-CAGGATTAGATACCCTGGTAGTCCACGC-3ʹ 5ʹ-GACGGGCGGTGTGTACAAGGCCCGGGAACG-3’	Universal (16S rDNA) primers	625 bp	b

a-Siqueira et al. 2004 [14], b-Goncharoff et al. 1993 [15]

### Generation of a cured derivative strain of lysogenic E. faecalis strain TUSoD11

In a previous communication we reported our generation and isolation of a cured *E. faecalis* strain [16]. Briefly, allelic exchange mutagenesis was employed to delete a module of six lysogeny-related genes and insert a selectable antibiotic resistance gene (erythromycin) into the ɸEf11 prophage of lysogenic *E. faecalis* TUSoD11. PCR screening of the recombinant transformant clones selected on erythromycin plates confirmed the absence of the targeted prophage genes in the cells of the recovered colonies. Surprisingly, in addition to the deletion of the six genes of the targeted lysogeny gene module, the cells of a few of the recovered colonies also lacked any other of the ɸEf11 prophage genes, for which they were screened, as well. Because the phage ɸEf11 genome is circularly permuted, deletion of the entire prophage from the TUSoD11 chromosome could have occurred through homologous recombination between the gene exchange vector that was used and homologous regions that could be positioned at either end of the ɸEf11 prophage. PCR screening was conducted using ɸEf11 prophage-specific primers and template DNA from presumptive recombinant clones selected on the antibiotic (erythromycin) plates. Those clones, no longer possessing any detectable ɸEf11 prophage genes, were considered cured of the prophage, and designated *E. faecalis* TUSoD11(ΔɸEf11). By this process we have obtained the isogenic pair of lysogenic and non-lysogenic *E. faecalis* strains [TUSoD11 and TUSoD11(ΔɸEf11)], differing only in the presence or absence of the ɸEf11 prophage.

### Growth rate assay

Cultures of lysogenic *E. faecalis* TUSoD11 and its cured isogenic derivative TUSoD11(ΔɸEf11) were grown overnight at 37°C in BHI broth. Portions of each culture were inoculated into fresh BHI broth to produce suspensions having an OD_600_ of 0.1. Samples of each suspension were placed into wells of a flat-bottomed 96 well microtiter plate (200 μl/well). Control wells contained uninoculated BHI broth. The plate was loaded into a microplate reader (Synergy HT), and incubated at 37°C for 24 h, during which the OD_600_ of each well was measured. The result of triplicate assays was recorded.

### Biofilm assay

Biofilms were established and assayed colorimetrically as described by Knezevic and Petrovic [17]. Cultures of *E. faecalis* TUSoD11 and TUSoD11(ΔɸEf11) were grown overnight at 37°C in modified LB broth (0.5% yeast extract, 1.0% Tryptone, 1.0% NaCl, 0.5% glucose). Each culture was diluted to OD_600_ of 0.1 using modified LB broth. Samples of 200 μl of each culture were inoculated into the wells of a flat bottomed 96 well microtiter plate. After incubation at 37°C for 48 h, the medium and planktonic cells in each well were removed and the wells were washed twice with PBS (0.072% Na_2_PO_4_, 0.021% KH_2_PO_4_, 0.765% NaCl, pH 7.2). The attached cells in each well were left to air dry, and then fixed by incubation in absolute methanol (200 μl per well) for 15 min. The fixative was then removed, the wells were allowed to air dry, and then crystal violet (0.4%) was added (200 μl per well). The stain was removed after 15 min and the plate was washed under a stream of tap water. After allowing the wells to air dry, 200 μl of 33% acetic acid was added to each well and, after 20 min, the OD_595_ of each well was read in a microplate reader (Synergy HT). Control wells were prepared using uninoculated modified LB broth. The results are the mean of five replicate cultures (± SE).

Additional biofilm assays were conducted following procedures modified from Merritt et al. [18]. Here, the colony forming units (CFUs) recovered from biofilms were directly enumerated by plating onto an agar medium. Briefly, biofilms of *E. faecalis* TUSoD11 or TUSoD11(ΔɸEf11) were established on sterilized circular (12 mm diameter) glass cover slips placed in wells of a 24 well plate. The culture medium, containing the planktonic cells, was removed and the wells containing the biofilm-coated cover slips were washed six times with 2 ml of sterile PBS. Each cover slip was then aseptically transferred to a sterile glass tube containing 4 ml of PBS whereupon it was sonicated (MSE, Soniprep 150 plus) for 8 sec at ~ 50% amplitude (≈ 7microns) and a power output of ~ 5 watts. Each sonicated cover slip (in 4 ml PBS) was vigorously vortexed for 5 sec, and the titer of the resulting *E. faecalis* suspension was determined by plating dilutions onto plates of Thallous Acetate Agar Medium, which is selective for enterococci [19].

### Plate lysis and turbidity reduction assays for detection of sensitivity to bacteriophage ɸEf11 endolysin

In the course of a productive infection, many bacteriophages (phages) synthesize muralytic enzymes (endolysins) to lyse the infected host cell and enable the release of the progeny virions. The external application of endolysins to strains of most (Gram positive) bacteria will also cause cell wall degradation and result in cell lysis from without. Previously, we isolated and characterized an endolysin produced by *E. faecalis* bacteriophage ɸEf11 [20]. Preparations of this endolysin (ORF28 endolysin) were used to test the sensitivity of the isogenic pair of lysogenic (TUSoD11) and cured [TUSoD11(ΔɸEf11)] *E. faecalis* strains.

For plate lysis assays, 0.1 ml of an overnight BHI broth culture of each of the two paired *E. faecalis* strains was inoculated into 3 ml of molten (45°C) soft agar (BHI broth containing 0.7% agar). This mixture was rapidly poured into plates containing a solid layer of BHI agar (BHI broth containing 1.5% agar), and allowed to solidify and air dry. The endolysin preparation (3 μl) was then spotted onto the center of the solidified soft agar layer, and this was allowed to air dry. The plates were then incubated at 37°C overnight, whereupon they were inspected for clear zones in the bacterial lawn where the spots were originally placed, indicating lytic activity.

For turbidity reduction assays, overnight 10 ml BHI broth cultures of the lysogenic (TUSoD11) and isogenic cured strain [TUSoD11(ΔɸEf11)] of *E. faecalis* were grown, and the cells were collected by centrifugation (7,500 x g x 10 min). The cells were resuspended in 5 ml of PBS, and 1.5 ml of this suspension was transferred into sterile, clear tubes. Each tube then received 30 μl of a filter sterilized preparation of the phage ɸEf11 ORF28 endolysin [20]. Control tubes received 30 μl of PBS instead of the endolysin. The tubes were incubated at 37°C and observed for changes in turbidity.

## Results

### Specificity of ɸEf11 F/ɸEf11 R primer set

**Figure 2.**
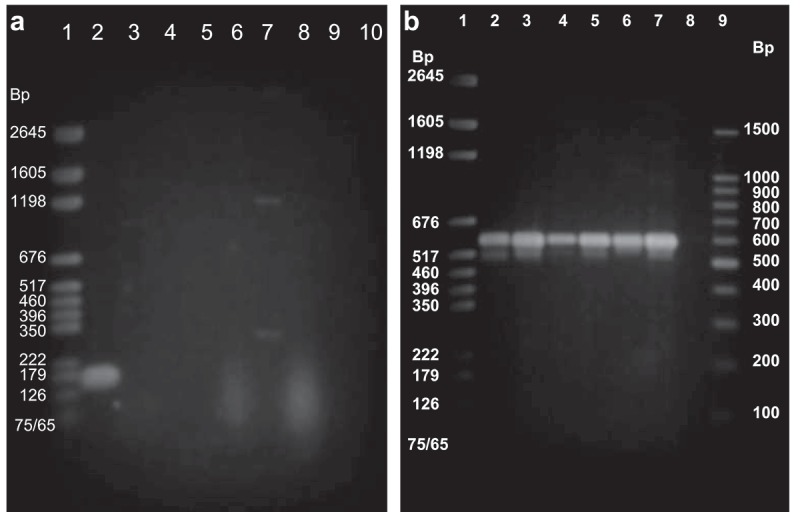
(a). Specificity of ORF43 primers for ɸEf11prophage. PCR reactions performed with ϕEf11F and ϕEf11R primers and template DNA from positive (lane **2**) and negative (lanes **3**–**10**) controls. Lanes: **1**, Bench Top Marker (Promega); **2**, *E. faecalis* TUSoD 11 (positive control); **3**, *E. faecalis* JH2-2 (negative control); **4**, *S. mutans*; **5**, *S. aureus*; **6**, *S. sanguinis*; **7**, *F. magna*; **8**, *C. perfringens*; **9**, *A. israelii*; **10**, *E. lentum*. Note that the 165 bp ϕEf11 bacteriophage-specific amplicon was only produced in the reaction containing template DNA from *E. faecalis* TUSoD 11 (lane **2**) that is known to harbor the ϕEf11 bacteriophage genome. (b). Presence of template DNA from each bacterial species in PCRs. PCR reactions performed with universal primers (RRN4 and RRN5) and template DNA from *E. faecalis* TUSoD11 (lanes 2, 3), *S. mutans* (lanes 4,5), *F. magna* (lanes 6,7). Lane 8 blank. Lane 1, Bench Top Marker (Promega), Lane 9, 100 bp ladder. Amplicons of the expected size (625 bp) confirmed presence of bacterial template DNA in each of the PCRs.

**Figure 3.**
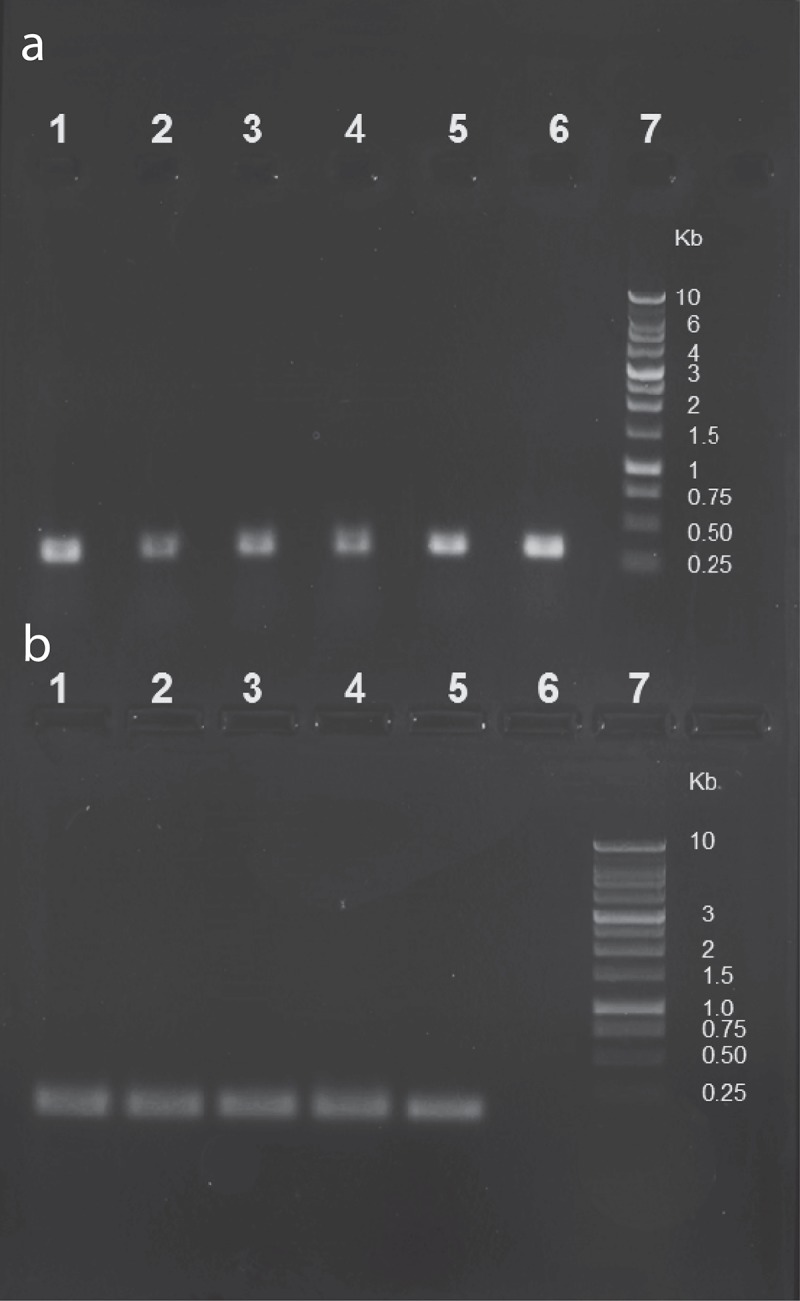
Presence of ɸEf11 prophage in oral *E. faecalis* strains. PCR reactions performed with (a) *E. faecalis*-specific primers (IF and 1R), or (b) ɸEf11 ORF 43-specific primers (ɸEf11F and ɸEf11R). Lanes: 1, *E. faecalis* GS25; 2, *E. faecalis* GS29; 3, *E. faecalis* GS33; 4, *E. faecalis* AA-OR34; 5, *E. faecalis* TUSoD11 (positive control); 6, *E. faecalis* JH2-2 (negative control); 7, Bench Top 1kb DNA ladder (Promega). 310 bp amplicons in A indicate the presence of *E. faecalis* DNA from each of the strains tested, and the 165 bp amplicons in B indicate the presence of phage ɸEf11 ORF43 in the DNA from each the same strains (with the exception of JH2-2, negative control).

TUSoD11 is the lysogenic *E. faecalis* strain from which phage ɸEf11 was originally induced and isolated [12]. Consequently we considered the TUSoD11 DNA to be a positive control for the presence of the phage ɸEf11 ORF43 sequence. *E. faecalis* strain JH2-2 supports lytic infection by phage ɸEf11 [12], and therefore, by reason of superinfection exclusion, this strain would not be expected to harbor the phage ɸEf11 genome. ORF43-specific primers produced an amplicon of the predicted size (165 bp) when used in PCRs with template DNA from *E. faecalis* TUSoD11 (Figure 2(a), lane 2). Furthermore, no amplicon of the predicted size was produced using the ORF43-specific primers in PCRs with templates from *E. faecalis* JH2-2 (Figure 2(a), lane 3), or any of the unrelated bacterial species tested (Figure 2(a), lanes 4–10). In contrast, PCRs using template from JH2-2 or any of the unrelated bacterial species with either *E. faecalis*-specific primers 1F and 1R (for JH2-2) or universal primers RRN4 and RRN5 (for the unrelated bacterial species) *did* yield amplicons of the expected size, ≈ 310 bp and ≈ 625 bp, respectively (Figures 2(b), 3(a)). These results demonstrate the specificity of the ɸEf11F/ɸEf11R primer set for the ɸEf11 ORF43 DNA sequence.

### Prevalence of ORF43 among 61 oral E. faecalis strains

We next used the ORF43-specific primers in PCRs with template DNA from 61 *E. faecalis* strains isolated from the human oral cavity. An example of the results from these reactions can be seen in Figure 3(b) where five of the *E. faecalis* strains tested produced amplicons of the predicted size. All in all, 19 (31%) of the 61 oral *E. faecalis* strains were found to be positive for the ɸEf11 ORF43 DNA sequence (Table 1).

### Cured, recombinant clones of TUSoD11 were generated lacking ɸEf11 prophage genes

**Figure 4.**
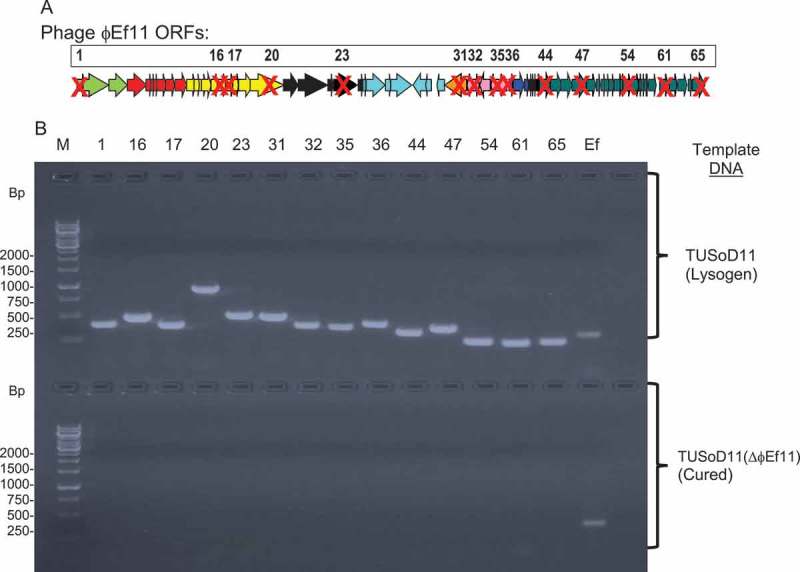
Deletion of ɸEf11 genes from cured derivative of *E. faecalis* strain TUSoD11(ΔɸEf11). (a). Representation of phage ɸEf11 genome (ORFs1-65). Packaging, Head morphogenesis, Tail morphogenesis, Lysis, Integration, Lysogeny establishment and maintenance, Lytic cycle regulation, DNA replication and modification. ORFs labeled **X** could not be detected in the cured strain. (b). PCR Screening of recombinant TUSoD11 clone for the presence of prophage ɸEf11 genes. AGE analysis of PCR amplicons produced with ɸEf11 ORF-specific primers (ORFs 1, 16, 17, 20, 23, 31, 32, 35, 36, 44, 47, 54, 61 and 65) and templates from either *E. faecalis* TUSoD11 (lysogenic strain) or TUSoD11(ΔɸEf11)(cured strain). Lane numbers refer to ORF specificity of primer set. M = molecular mass standard (GeneRuler1kb DNA ladder), Ef = *E. faecalis*-specific primer set. Amplicons in upper half of the figure were generated using lysogenic *E. faecalis* TUSoD11 template, PCRs in lower half of the figure were conducted with template from (cured) TUSoD11(ΔɸEf11).

Primer sets specific for numerous ɸEf11 prophage genes were used in PCR to screen for the presence of the prophage in TUSoD11 clones that had been transformed with a gene exchange vector. As can be seen in the example shown in Figure 4, clones were identified in which none of the 14 ɸEf11 prophage genes examined could be detected by PCR. These clones were considered to be cured, and were designated TUSoD11(ΔɸEf11).

### Lysogeny promoted the maintenance of a higher cell density relative to the cured strain

**Figure 5.**
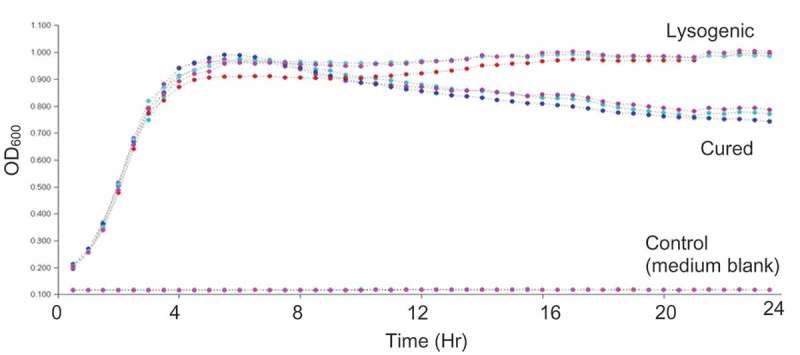
Comparative growth curves of Lysogenic (TUSoD11) and Cured [TUSoD11(ΔɸEf11)] strains of *E. faecalis*. Each strain was grown in triplicate.

Growth curves of the lysogenic strain (TUSoD11) and the cured strain [TUSoD11(ΔɸEf11)], were compared. As seen in Figure 5, the growth rates of the two strains were quite similar for the first 12 h of incubation, however after 12 h, the cell density of the lysogenic strain suspension was maintained, and even increased over the next 12 h. In contrast, the cell density of the suspension of the cured strain steadily decreased between 12 and 24 h of incubation.

### Biofilm formation is enhanced in the lysogen compared to the cured E. faecalis strain

**Figure 6.**
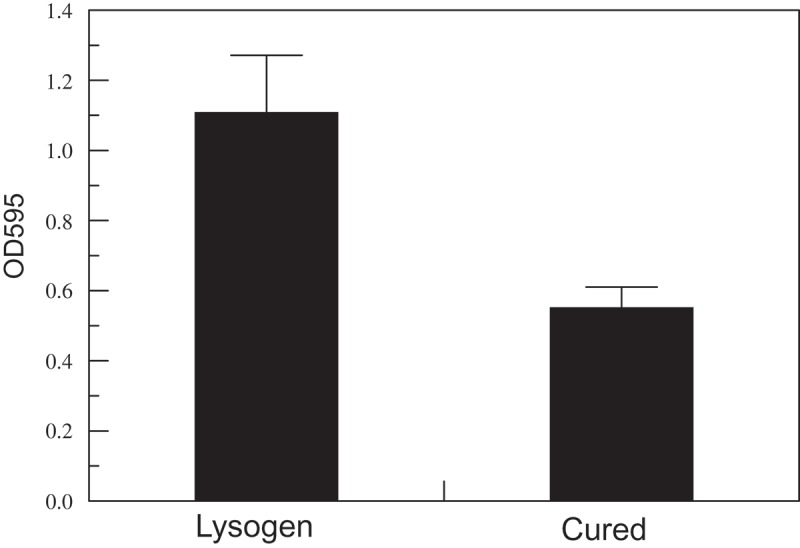
Biofilm assay by crystal violet staining. Colorimetric measurement of biofilm formation by lysogenic (TUSoD11) and cured [TUSoD11(ΔɸEf11)] *E. faecalis* strains. Biofilms formed by lysogenic and cured strains were stained with crystal violet. Values are the mean of five replicate cultures ±SE.

**Figure 7.**
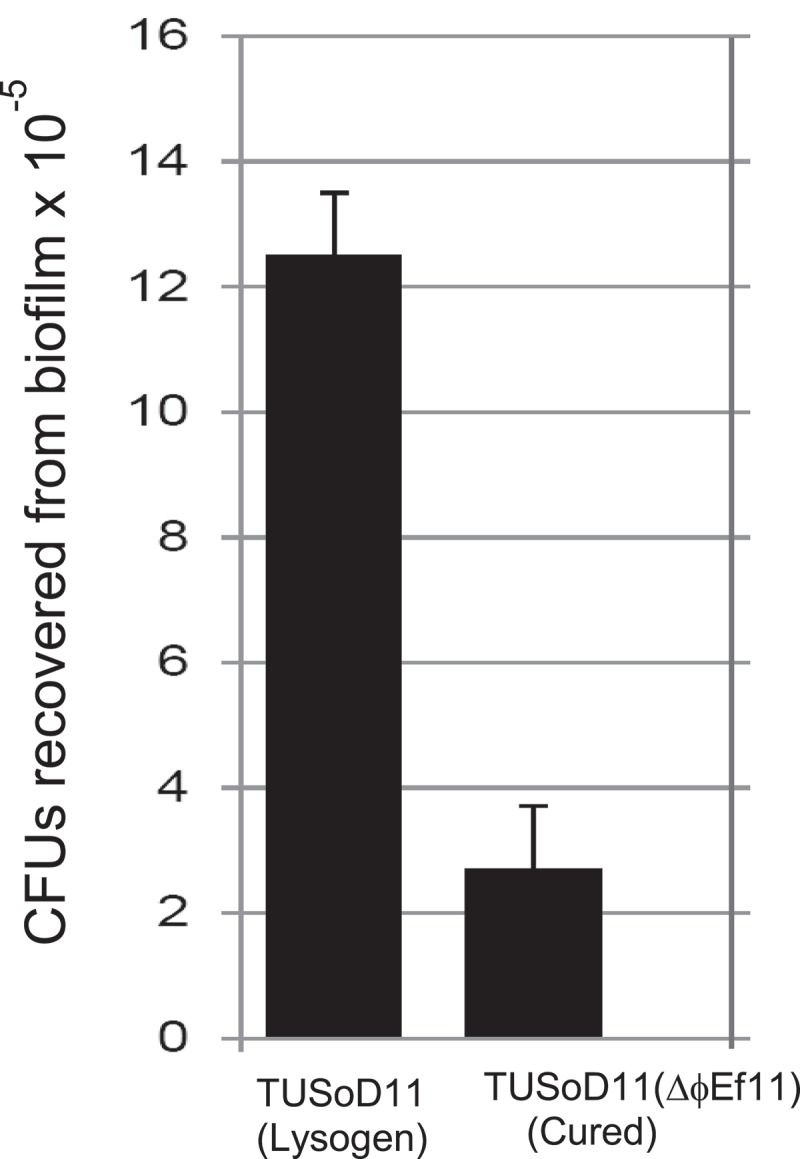
Biofilm assay by direct bacterial enumeration. Colony Forming Units (CFUs) recovered from biofilms produced by lysogenic (TUSoD11) and cured [TUSoD11(ΔɸEf11)] *E. faecalis* strains. Biofilms formed by lysogenic and cured strains were sonicated, and the suspended cells were recovered and tittered. The values are mean of three replicate cultures ±SE.

In comparison with the cured *E. faecalis* strain [TUSoD11(ΔɸEf11)], the isogenic, lysogenic strain (TUSoD11) formed approximately 50% more biofilm (Figure 6). Furthermore, enumeration of the bacteria recovered from biofilms disclosed that the lysogenic *E. faecalis* strain (TUSoD11) yielded approximately six times the colony forming unit count of its isogenic cured counterpart [TUSoD11(ΔɸEf11)] (Figure 7). This suggests that the enhanced biofilm formation produced by the lysogenic strain observed by crystal violet staining, is due, at least in part, to a higher bacterial population.

### The presence of a prophage renders the lysogenic cell resistant to the lytic action of an externally applied bacteriophage endolysin

**Figure 8.**
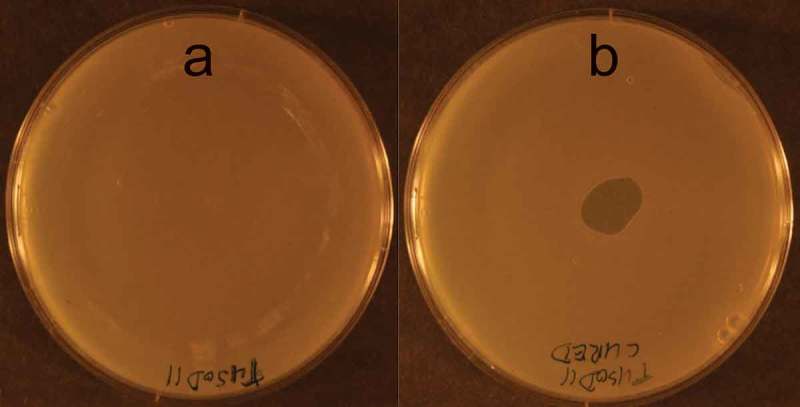
Plate lysis assay for sensitivity of *E. faecalis* strains to phage ɸEf11 endolysin. Layers of either (a) *E. faecalis* TUSoD11 (lysogen) or (b) TUSoD11(ΔɸEf11) (cured) were prepared. A drop of endolysin suspension was placed on the center of each layer, and the plates were incubated at 37°C overnight. Note the lytic zone in the layer of the cured strain.

**Figure 9.**
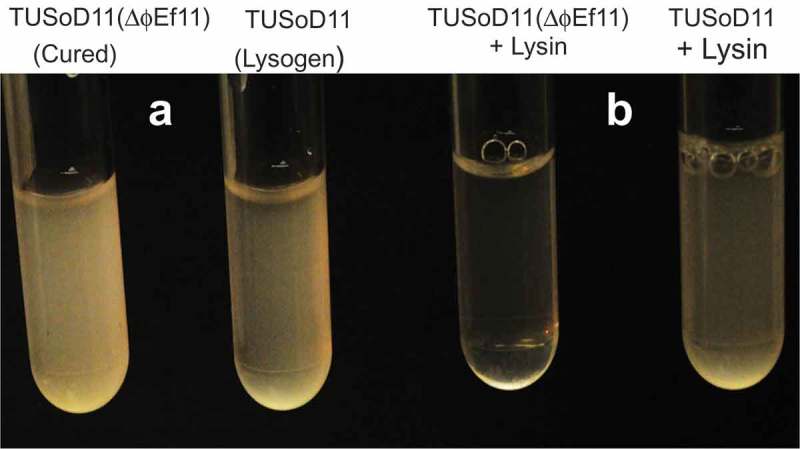
Effect of phage endolysin on suspensions of lysogenic (TUSoD11) and cured [TUSoD11(ΔɸEf11)] *E. faecalis* strains. (a). Suspensions at 0 time. (b). Suspensions after incubation with the endolysin for 30 min.

Spotting 3 μl of the phage ɸEf11 endolysin preparation onto a lawn of the cured *E. faecalis* strain [TUSoD11(ΔɸEf11)] produced a clear lytic zone, whereas no such effect was seen in a lawn of the lysogenic strain, TUSoD11 (Figure 8) Furthermore, addition of the ɸEf11 ORF28 endolysin to a suspension of the cured *E. faecalis* strain [TUSoD11(ΔɸEf11)] resulted in a rapid and pronounced clearing (Figure 9). In contrast, a suspension of the lysogenic strain (TUSoD11) exhibited no overt change following the addition of the endolysin (Figure 9).

## Discussion

The development and introduction of culture-independent, molecular methods of microbial detection and identification has resulted in a more thorough appreciation of the microbial diversity throughout the human body. Compared to the cultural methods previously used, *16S RNA* gene amplification and sequencing can provide a more complete assessment of microbial population composition. The microbiomes of the oral cavity [21] and several individual ecological niches within the oral cavity, such as the saliva [22–24], the periodontal pocket [25–27], the dorsum of the tongue [28] and the infected root canal [29–31] have been explored using this technique. However, it is also true that *16S RNA* gene sequencing technology is not well suited to provide information on intraspecies strain variation. This is of some concern since it is well established that virulence properties of many pathogenic bacteria are due to the uptake of exogenous genetic information, such as plasmids and phage genomes, thereby modifying the genome of individual strains of a given species. Such modifications could go undetected by a simple enumeration of species present in a given microbiome.

Other studies, using pyrosequencing methods have described the virome of the human oral (salivary) environment [32]. This study determined that > 99% of the assembled contigs were homologous to bacteriophage sequences, suggesting that the vast majority of the human salivary virome was composed of bacteriophages. The metabolic gene profile of the viral DNA population was dominated by genes that were identified as coding for functions related to nucleic acid metabolism and virulence factors. In addition, approximately 10% of the viral contigs had integrase homologs, suggesting a temperate bacteriophage origin of a substantial portion of the oral virome genome. However, these data do not provide information on the proportion of the oral (bacterial) microbiome that has actually been influenced by the horizontal gene transmission of these viral genes through lysogeny. In an analysis of the salivary bacteriophage transcriptome in health versus periodontal disease, it was found that many bacteriophage genes are expressed in the oral cavity in both health and disease [33], however, here again, it cannot be ascertained to what extent the resident bacterial population is chronically infected by a virus, or how the host bacteria are altered (in the case of lysogenic infection). Here, we identified a strain variability within one species of oral bacteria *(E. faecalis*) in terms of the presence or absence of a prophage in the bacterial genome, and illustrate some of the ways in which this variation (lysogeny) has impacted the properties of the host cell.

We used PCR to detect an ɸEf11 phage-specific sequence (ORF43) in a panel of oral *E. faecalis* strains. The ORF43 sequence was selected as a phage ɸEf11 indicator due to its uniqueness among all the sequences searched in databases. The specificity of the ORF43 PCR primer set was validated using negative controls including template DNA from unrelated bacterial species, and *E. faecalis* strains, such as JH2-2, that supported lytic infection by phage ɸEf11. Lysogenic *E. faecalis* strains harboring an ɸEf11 prophage do not support lytic infection by phage ɸEf11 due to superinfection immunity. In contrast, template from a positive control strain, *E. faecalis* TUSoD11, the strain from which phage ɸEf11 was originally induced, did produce an amplicon of the predicted size (165 bp) when used in PCR with the ORF43 primers. Using these primers, we found that lysogeny among the oral *E. faecalis* strains tested was fairly common, with 19 out of the 61 (31%) strains displaying evidence of harboring an ɸEf11 prophage. Furthermore, it is likely that this incidence of lysogeny (31%) is an underestimation of the prevalence of lysogeny in oral *E. faecalis* strains since our procedures would only allow the detection of ɸEf11 prophages in the strains examined, and there have been several different *E. faecalis* phages detected in human oral samples [34,35]. Lysogens containing these prophages would not have been detected in our study.

The prevalence of lysogeny among our panel of oral *E. faecalis* strains is not unexpected. Lysogeny is widespread in nature and can be anticipated to be a common feature of oral bacterial strains, as suggested by the findings of the previously mentioned study of human salivary virome [32]. It has been reported that approximately half of all sequenced bacterial genomes contain one or more prophages [36–39]. The high incidence of putative integrase-related sequences in the salivary virome reported by Pride et al. [32] suggests the likelihood that the oral cavity harbors a high proportion of temperate bacteriophages potentially capable of producing lysogenic infections. In this regard, we previously demonstrated that lysogeny was widespread in strains of another oral bacterium, *Aggregatibacter (Actinobacillus) actinomycetemcomitans*, a species associated with aggressive localized periodontitis [40]. Among the 42 *A. actinomycetemcomitans* strains tested 14 (34%) were found to be lysogenic.

Lysogeny often results in altered phenotypic properties of the host bacteria [41–43]. These alterations include: resistance to superinfection by subsequent phages, enhanced virulence, and increased fitness (improved ability to outcompete nonlysogenic strains). Alterations can be due to expression of prophage genes in the lysogen [44] or to modifications of host gene expression as a consequence of the insertion or excision of the prophage into or out of the bacterial chromosome [45]. In either case, the new phenotype may provide a selective advantage for the lysogen. Our results suggest that lysogenic infection of *E. faecalis* by phage ɸEf11 results in an elevated persistent yield, a denser biofilm formation, and resistance to a phage-coded lytic enzyme. The increase in growth (higher cell density maintained during stationary phage) seen in the lysogenic *E. faecalis* strain is in agreement with previous studies reporting that the presence of a prophage confers the ability for the lysogen to grow at higher rates and produce higher yields in stationary growth phase [39]. Lysogens of *Escherichia coli* [46–48] and *Streptococcus suis* [49] have been shown to grow more rapidly and maintain higher stationary phase titers than their nonlysogenic counterparts. Similarly, our finding that lysogeny increases biofilm production in *E. faecalis* is in accord with several previous studies demonstrating the biofilm-promoting effect of the presence of a prophage [48,50–52]. Both of these properties may play a role in the fitness of the *E. faecalis* strain to compete in the oral environment.

The differential sensitivity to the phage endolysin between the lysogenic and cured *E. faecalis* strains is somewhat surprising. The endolysin is a murein hydrolase that is produced by a phage in the course of a lytic infection. It is required by the virus in order to lyse the infected cell and permit the release of the progeny virions. Therefore, it might be expected that the lysogenic strain (TUSoD11), harboring the ɸEf11 prophage, should be sensitive to the endolysin. The apparent lack of sensitivity of the lysogenic strain to the endolysin might be due to the fact that in our assay, the endolysin was applied externally to the cell layer, whereas during lytic infection, the source of the endolysin is internal, from within the infected cell. It is conceivable that lysogeny results in surface modification of the infected cell, rendering it resistant or inaccessible to externally applied endolysin. Prophage-mediated cell surface modification has been well documented for many other phage-host systems [53–66]. Cell wall polysaccharides and capsules are known to be produced by many *E. faecalis* strains [67–69], especially those of oral origin [70–72]. If these cell-surface components are modified due to lysogenic conversion, and they no longer serve a vital function needed for lysin activity, then as we observed with lysogenic strain (TUSoD11), no lytic activity would be detected. The cured strain [TUSoD11(ΔɸEf11)], lacking any phage-mediated cell surface modification, would (and did) remain sensitive to the lytic effects of the endolysin. It is not immediately clear whether this explanation is accurate for the ɸEf11 lysogens. Although many (33/65) of the bacteriophage ɸEf11 genes have been annotated [13], none of these appear to be related to cell surface polysaccharide or capsule modification. It is possible that one of the remaining uncharacterized phage genes plays a role in modification of the host cell, however this remains to be determined. Further studies are needed to determine the capsular status of both the lysogenic (TUSoD11), and cured TUSoD11(ΔɸEf11)], strains.

While the present investigation compares properties of pure/individual cultures of an isogenic pair of lysogenic and cured *E. faecalis* strains *in vitro, in vivo* lysogeny may impact mixed cultures as well. Phage produced as a result of induction of lysogenic strains may infect and kill susceptible nonlysogenic strains, resulting in the competitive advantage of the remaining uninduced lysogens, which are immune to superinfection. If a similar relationship exists for the many other bacterial species of the oral microbiome, then the oral microbial composition may be markedly shaped by lysogeny, and the bacterial viruses produced by the induction of lysogens.

This investigation demonstrated the impact of lysogeny on just three phenotypic characteristics of *E. faecalis*. Clearly, there are numerous other features that are known to be modifiable due to lysogenic conversion in other bacterial species [3,43,45,73–76] and many of these deserve to be examined in relation to *E. faecalis* infection. To date, relatively few studies have been conducted to identify phage-mediated modifications of *E. faecalis*. Two such phenotypic modifications of *E. faecalis* due to lysogenic conversion that have previously been demonstrated are the production of homologs of platelet binding proteins PblA/PblB of *Streptococcus mitis* phage SM1 [77] and enhanced intestinal colonization [78]. *E. faecalis* lysogens harboring prophages encoding PblA/PblB homologs exhibit enhanced platelet adherence compared to strains lacking these prophages [77]. Mixed infection by lysogenic and phage-sensitive *E. faecalis* strains in mice resulted in a 1.5 fold enrichment of the lysogen in the colon [78]. In light of the widespread incidence of lysogeny in oral *E. faecalis* strains, further studies are needed to evaluate the potential modulation of other properties due to the presence of a prophage. Furthermore, to more thoroughly understand the factors driving oral microbiome establishment and pathogenic potential, more investigation is needed into the prevalence and significance of lysogeny among the many other members of this microbial community.
